# HIV-1 Subtype and Reverse Transcriptase Genotype: Role for Geographical Location and Founder Effects

**DOI:** 10.1371/journal.pmed.0030540

**Published:** 2006-12-26

**Authors:** Akinyemi I Ojesina, Phyllis J Kanki

In a previous issue of *PLoS Medicine*, Kantor et al. [1] published very impressive results from an international multicenter collaboration in which they reported significant overlap between subtype B drug resistance mutations and mutations associated with at least one non-B isolate in HIV-1 reverse transcriptase (RT) and protease. This information is very useful for planning large-scale global surveys for antiretroviral drug resistance.

The authors described subtype-specific polymorphisms as mutations that were significantly more prevalent in each non-B subtype than in subtype B viruses from untreated persons. Amongst these was an A98S mutation in HIV-1 reverse transcriptase, which was described as a subtype G–specific polymorphism.

In Nigeria, the predominant variants of HIV-1 are the circulating recombinant form CRF02_AG and subtype G. The 98S polymorphism was not found in 35 of 35 sequences obtained from a group of HIV-1-infected drug-naïve Nigerians, and the consensus at this position was A98 [2].

Kantor et al. [1; Table 1 and Figure 4] describe how 207 out of 294 (70%) of the subtype G samples were obtained from Portugal and Spain, while approximately half of the subtype G samples had polymorphisms (with respect to subtype B consensus) at RT position 98. We hypothesized that a significant correlation exists between the samples from Portugal and Spain and the presence of the A98S polymorphism in HIV-1 reverse transcriptase reported by the authors. It is however possible that this polymorphism is similarly prevalent in subtype G sequences from other countries.

We therefore interrogated the Stanford HIV Drug Resistance Database [3], where the updated results from this international collaboration maintain detailed RT mutation data for subtype G isolates. The output has detailed information on GenBank accession numbers and publication data, from which we deduced the country of sampling. In all, amino acid residue information for RT position 98 was available for 500 subtype G isolates from both drug-naïve and treated persons: 351 isolates (70%) with the A98S polymorphism, 143 isolates (29%) with the wild type A98A residue, and six isolates (1%) with the A98G mutation.

In order to determine if A98S is a subtype G-specific polymorphism, only the 165 isolates obtained from drug-naïve persons with either A98S or A98A were used in this analysis. 57 (34.5%) of these selected isolates were from Portugal and Spain. 51 (89.5%) of the isolates from Portugal and Spain had the A98S polymorphism, compared with only 10 (9.3%) of the 108 isolates from other countries combined. Therefore, a very strong association exists between the presence of the A98S polymorphism and sampling from Portugal and Spain (*p* is less than 0.0001). This suggests that the A98S polymorphism in HIV-1 subtype G is not subtype-specific, but may be the consensus amino acid residue for samples from Portugal and Spain. Indeed, this polymorphism has been previously reported as being unique to samples from these countries [4].

Considering that various HIV-1 variants have geographical bias [5], it is possible that patterns of subtype-specific polymorphisms may be differentially predominant in certain geographical locations with respect to others. The presence of specific mutations in drug-naïve individuals may influence decisions on the choice of therapeutic regimens [6]. Therefore, the description of subtype-specific polymorphisms without stratifying by geographical location, or controlling for the role of closely related founder viruses, may result in misleading generalizations. Examination of the role of the source of samples in multicenter studies may be pertinent in assigning the tag of subtype-specific polymorphisms, especially for HIV-1 variants with significant transcontinental distribution, for example, HIV-1 subtypes C and G.
